# Characterization and Comparison of Fumonisin B_1_-Protein Conjugates by Six Methods

**DOI:** 10.3390/ijms13010084

**Published:** 2011-12-22

**Authors:** Ying Wang, Cheng-Hua He, Hao Zheng, Hai-Bin Zhang

**Affiliations:** College of Veterinary Medicine, Nanjing Agricultural University, Nanjing, 210095, China; E-Mails: wangying0624@hotmail.com (Y.W.); hechenghua@njau.edu.cn (C.-H.H.); zebeihao@yahoo.com.cn (H.Z.)

**Keywords:** Fumonisin B_1_, conjugated antigen, carrier protein, molar ratio, antibody

## Abstract

In order to generate an antibody against a small hapten molecule, the hapten is cross-linked with carrier protein to make it immunogenic. In this study, the hapten (Fumonisin B_1_, FB_1_) was coupled to ovalbumin (OVA) and bovine serum albumin (BSA), respectively by a short cross-linker reagent (glutaraldehyde, GA). To develop a technique for detecting the conjugation, the hapten-protein conjugates (FB_1_-OVA and FB_1_-BSA) were characterized thoroughly by ultraviolet (UV) spectroscopy, Fourier transform infrared (FT-IR) spectroscopy, gel electrophoresis and matrix-assisted laser desorption ionization time-of-flight mass spectrometry (MALDI-TOF-MS), respectively. The molecular weights of FB_1_-BSA and FB_1_-OVA were 74,355.301 Da and 48,009.212 Da, respectively determined by the method of MALDI-TOF-MS. The molecular coupling ratios were 11 and 5 in FB_1_-BSA and FB_1_-OVA, respectively. In this experiment, MALDI-TOF-MS was selected as the most efficient method to evaluate the cross-linking effect and calculate the molecular coupling ratio.

## 1. Introduction

Fumonisins, which assume significance due to their deleterious effects on animals, are a group of potent, carcinogenic, hepatotoxic and nephrotoxic secondary metabolites produced mainly by *Fusarium verticillioides* (formerly *Fusarium moniliforme* Sheldon) and *Fusarium proliferatum* [1–3]. Fumonisin B_1_ (FB_1_), the most common and highest toxic of fumonisins species, is the focus of governments and scientists throughout the world due to the strong toxicity and potent carcinogenicity shown in animal studies [4]. FB_1_ is hepatotoxic and nephrotoxic and it can cause serious diseases in equine species, swine and rodents. Furthermore, FB_1_ may be correlated to the high incidence of human esophageal cancer [5–7]. Due to its widespread existence in agriculture, the pollution in cereal and products has been reported, which may cause potential health hazards [8–10]. Switzerland has proposed legislation for FB_1_; the limit was determined as 1000 μg/kg [11,12]. The Food and Drug Administration (FDA) has issued maximum residue limits in maize, maize byproducts in food and animal feeds, which are 2000–4000 and 5000–10,0000 μg/kg total fumonisins (FB_1_ + FB_2_ + FB_3_) for human foods and animal feeds, respectively [13,14]. The Commission regulation of European Community (EC) has regulated the maximum levels of FB_1_ + FB_2_ for certain contaminants in foodstuffs vary from 200 to 4000 μg/kg depending upon the intended use of the maize [14]. Thus, there are strong economic and safety reasons for establishing more sensitive, selective, inexpensive, large-scale and rapid analytical methods for routine screening of FB_1_. To date, the methods for detecting FB_1_ have been mainly based on instrumental and immunological methods [15–18]. These extensive investigations and applications have resulted in rapid progress of immunoassays for FB_1_ that have led to a great demand for specific antibodies.

**Figure 1:**
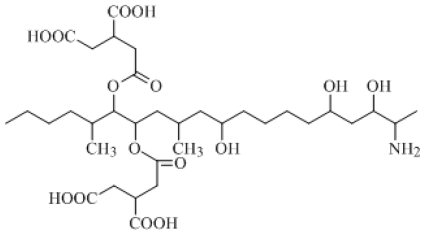
Constitutional formula of FB_1_.

The molecular formula of FB_1_ is C_34_H_59_NO_15_ and its formal name is 1,1′-[(1*S*,2*R*)-1-[(2*S*,4*R*,9*R*, 11*S*,12*S*)-12-amino-4,9,11-trihydroxy-2-methyltridecyl]-2-[(1*R*)-1-methylpentyl]-1,2-ethanediyl-1,2,3-(2*R*,2′*R*)-propanetricarboxylic acid ester. The constitutional formula of FB_1_ is shown in Figure 1.

Small molecules such as mycotoxins, pesticides, drugs, *etc*. are usually nonimmunogenic and hence do not elicit an immune response unless coupled with some macromolecules such as proteins [19]. The modification of these substances is therefore required in order for them to couple with macromolecules (carriers) to make a stable carrier-hapten complex [20]. Synthesis of hapten for linking with carrier protein is the most important aspect of specific antibody generation against a small molecule for immunoassay applications [21]. The most frequently used carrier proteins for conjugation are bovine serum albumin (BSA), ovalbumin (OVA), conalbumin (CONA), thyroglobulin (TG), immunoglobulin (Ig), fibrinogen, and keyhole limpet hemocyanin (KLH). To prepare an effective hapten-protein conjugate for the desired immune response, it is important to characterize the resulting hapten-protein conjugate to determine the hapten molecular coupling ratio on carrier protein (numbers of FB_1_ molecules per carrier) [22].

There has been significant progress in recent years in hapten-protein conjugates for the generation of anti-hapten antibodies with applications in immunoassays for small molecules [23–25]. Verification of the coupling reaction and determination of the molecular coupling ratio can be accomplished by ultraviolet (UV) spectroscopy scan, mainly by evaluating the available free amino groups before and after conjugation by radiolabeled haptens [26]. The characterization for conjugation by spectrophotometer method depends on the UV character of hapten and protein. However, it is difficult to determine the molecular coupling ratio of FB_1_ to carrier protein due to the lack of UV absorption groups in structure of FB_1_. It is necessary to develop a technique for determining the conjugation for FB_1_. The aim of the present study is to find an available method for identifying the conjugation of FB_1_ to carrier proteins and six commonly used methods are described and compared.

## 2. Results and Discussion

The functional group of the hapten governs the selection of the conjugation method to be employed. In this study, GA is utilized as a short cross-linker reagent to aid FB_1_ linking to BSA and OVA, respectively. This approach ensures stable cross-linking of hapten with protein along with acid amide and carbons bridge formation. The molecular coupling ratio of hapten per protein in the conjugate is an important parameter which generally defines the quality and quantity of antibody produced. A good antibody titer with higher specificity can be generated with an optimum number of haptens per protein molecule.

The characters of the new FB_1_-protein conjugates (FB_1_-OVA and FB_1_-BSA) are confirmed by ultraviolet (UV) spectroscopy, Fourier transform infrared (FT-IR) spectroscopy, nondenaturing agarose gel electrophoresis (N-AGE), nondenaturing polyacrylamide gel electrophoresis (N-PAGE), sodium dodecyl sulfate polyacrylamide gel electrophoresis (SDS-PAGE) and matrix-assisted laser desorption ionization time-of-flight mass spectrometry (MALDI-TOF-MS) analysis, respectively. In order to develop a technique for determining the conjugation, the performance of the six various methods are described and compared.

**Figure 2:**
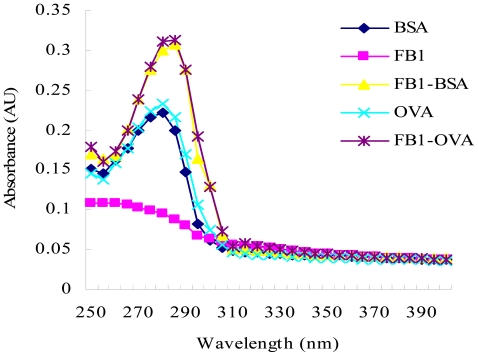
Comparative ultraviolet absorption spectra with conjugates and controls. One milligram of FB_1_ was dissolved in acetonitrile-water (50:50, v/v) (1.0 mg/mL) and the same concentrations of ovalbumin (OVA) and bovine serum albumin (BSA) were prepared. FB_1_-OVA and FB_1_-BSA were concentrated to the concentration of 1.0 mg/mL, respectively. One hundred microliters of the reaction mixture were injected into the UV apparatus. The scan range was from 250 nm to 400 nm.

The absorption spectra from UV spectroscopy experiments with FB_1_-BSA, FB_1_-OVA, and the controls (FB_1_, BSA and OVA) in the range of 250–400 nm are shown in Figure 2. In this case, the characteristic absorbance contribution of FB_1_ is not detected due to the lack of ultraviolet functional groups. The characteristic absorbance contribution of FB_1_-OVA and FB_1_-BSA are observed at 285 nm and 281 nm, respectively, whereas that contribution of the carrier protein (OVA or BSA) is observed at 280 nm. In the condition of the equivalent weight, there is a different absorbance between conjugate and carrier protein; the absorbance of conjugate is higher than the carrier protein. Theoretically, UV method is not used to identify FB_1_ conjugates due to the character of FB_1_. In this study, it is proved that the method of UV spectroscopy can be used to determine the course of conjugation by monitoring the relative shift in wavelength due to the contribution of the red shift and intensity of conjugate.

**Figure 3:**
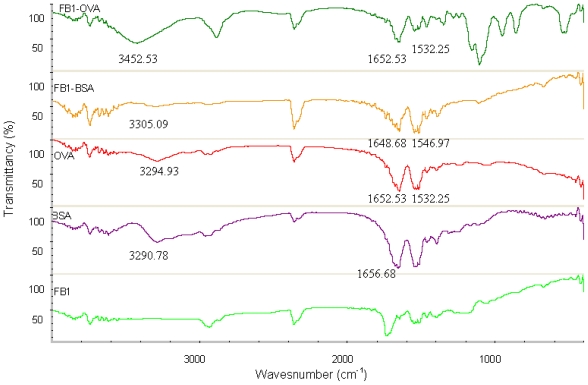
Fourier transform infrared (FT-IR) transmittance spectra of the conjugates and controls. One milligram of FB_1_ (OVA, BSA, FB_1_-OVA or FB_1_-BSA) was milled with KBr to form a very fine powder, respectively. This powder was then compressed into a thin pellet which could be analyzed using the intelligent FT-IR spectrometer. The scan range of 4000–400 cm^−1^. KBr was also transparent in the IR.

FT-IR spectroscopy is a measurement of wavelength and intensity of the absorption of IR radiation by a sample. The IR spectral data of high polymers are usually interpreted in terms of the vibrations of a structural repeat unit. The polypeptide and protein repeat units give rise to nine characteristic IR absorption bands, namely, amide A, B, and I–VII. Of these, the amide I and II bands are the two most prominent vibrational bands of the protein backbone. The most sensitive spectral region to the protein secondary structural components is the amide I band (1700–1600 cm^−1^), which is due almost entirely to the C=O stretch vibrations of the peptide linkages. The amide II band (1575–1480 cm^−1^), in contrast, derives mainly from in-plane NH bending and from the CN stretching vibration [27]. The characteristic IR groups of the FB_1_-BSA, FB_1_-OVA, BSA OVA and FB_1_ in the range of 4000–400 cm^−1^ are shown in Figure 3. There are the strong absorption from BSA and OVA, in the regions 3400–3300 cm^−1^, 1700–1600 cm^−1^ and 1575–1480 cm^−1^, which are the sensitive spectral region for Amide A, I and II of proteins. Compared with that of BSA, the characteristic absorption peaks of FB_1_-BSA could be found in the regions 3400–3300 cm^−1^, 1700–1600 cm^−1^ and 1575–1480 cm^−1^. The functional groups of OVA and BSA are contained in FB_1_-OVA and FB_1_-BSA, respectively. There is no strong absorption from FB_1_, in the regions 3400–3300 cm^−1^, 1700–1600 cm^−1^ and 1575–1480 cm^−1^, these spectral regions are particularly important for the study of proteins. It is proved that the conjugation of the hapten and proteins is successful by the similar absorption peak of conjugates and proteins. FT-IR method can be used to detect the cross-linking effect due to the apparent difference of specific spectra between FB_1_-protens and the controls.

**Figure 4:**
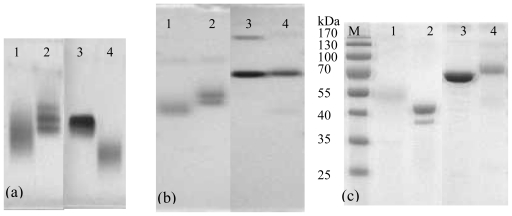
Gel electrophoresis of conjugates. (**a**) nondenaturing agarose gel electrophoresis (N-AGE); (**b**) nondenaturing polyacrylamide gel electrophoresis (N-PAGE) and (**c**) sodium dodecyl sulfate polyacrylamide gel electrophoresis (SDS-PAGE). Note: (1) FB_1_-OVA; (2) OVA; (3) BSA; (4) FB_1_-BSA and (M) protein marker. The concentration of protein standard (BSA or OVA) and conjugate (FB_1_-OVA or FB_1_-BSA) was diluted in dH_2_O to 0.2 mg/mL.

The gel electrophoresis of conjugates is shown in Figure 4. The pure proteins and conjugates in N-AGE and N-PAGE show well-defined bands, respectively. The band of FB_1_-OVA is moved further than OVA obviously in both N-AGE (Figure 4a) and N-PAGE (Figure 4b). Compared to that of BSA, the migration of FB_1_-BSA changes obviously in N-AGE, whereas it changes indistinctively in N-PAGE, which is probably due to the fact that the molecular weight of FB_1_-BSA is similar to that of BSA. For SDS-PAGE (Figure 4c), it is observed that the molecular weights of FB_1_-BSA and FB_1_-OVA are approx 70 kDa and 48 kDa, respectively. Although the electrophoresis analyses of conjugation cannot give definite information about the degree of the molecular coupling ratio, its main advantage is in detecting the course of cross-linked conjugation.

The optimum use of high grade of reagents along with thorough dialysis to remove unbound hapten and ion resulted in good coupling efficiency of hapten to protein as shown by the results of SDS-PAGE, N-AGE and N-PAGE and each conjugate generated well-defined band.

**Figure 5:**
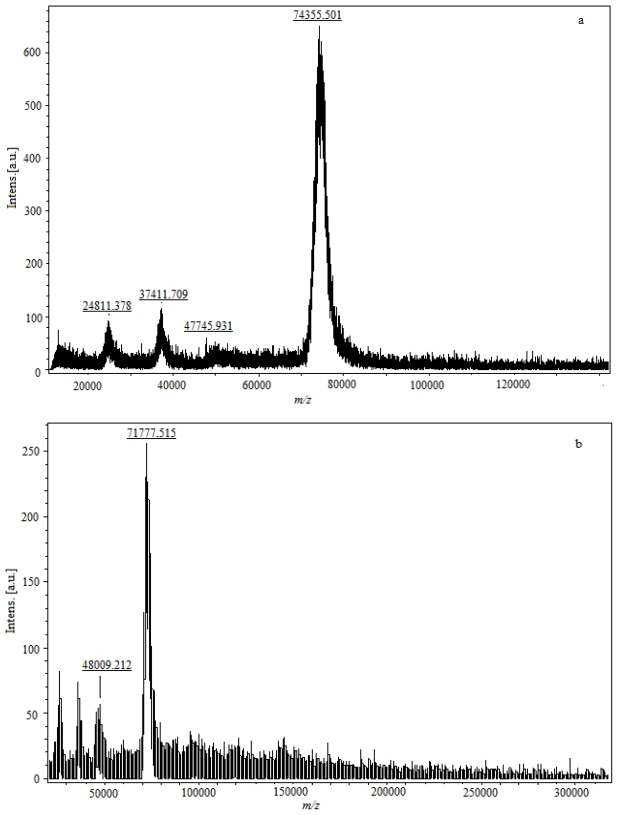

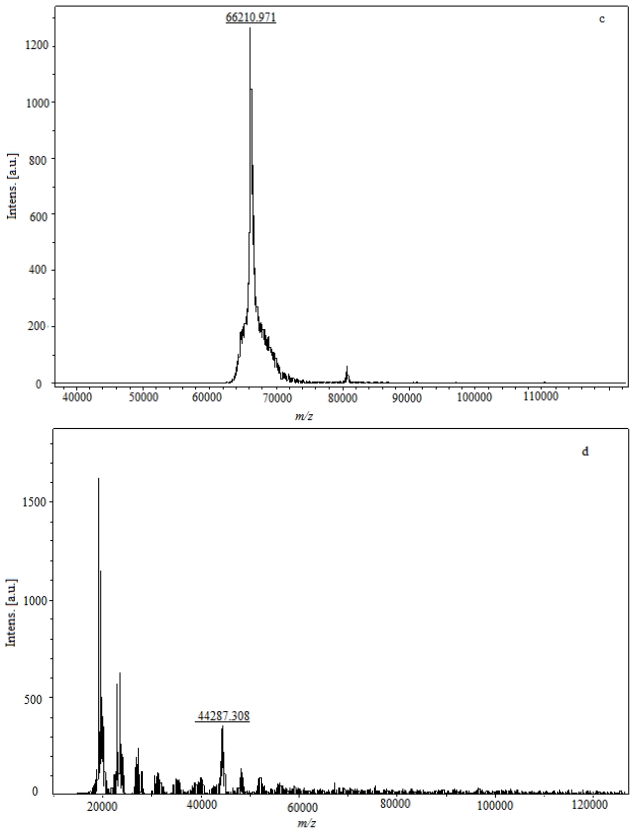
MALDI-TOF-MS of conjugates (FB_1_-OVA and FB_1_-BSA) (**a**) FB_1_-BSA; (**b**) FB_1_-OVA; (**c**) BSA; (**d**) OVA. The standard powder of controls (BSA, OVA) and lyophilized conjugates (FB_1_-OVA and FB_1_-BSA) were used for the scan.

Conjugation density for hapten resultes in a detectable increase in the molecular weight of the conjugate as determined by observing the peak shift of MALDI-TOF-MS (Figure 5). The parent molecular ion is formed by adding a charged species (usually a proton), signed as (M + H)^+^, and the *m/z* value of the molecular ion peak is approximately equal with the molecular weight of sample. The fragment ion is formed by adding multiple protons; an ion formed by adding single or multiple non-proton charging species; an ion formed by clustering of parent molecular species; or a combination of the latter, signed as (M + 2H)^+2^, (M + 3H)^+3^, (M + Na)^+^, (2M + H)^+^, (2M – H + 2Na)^+^, respectively. The incremental change in molecular weight due to incorporation of hapten molecules to protein corresponded to the number of hapten molecules per protein molecule. In Figure 5a, the *m/z* of the parent molecular ion is 74,355.301, and the *m/z* of 24,811.378, 37,411.709 are the fragment ions concomitantly. The weight of modified FB_1_-protein is significantly higher than the molecular weight of the native protein. The molecular weight of FB_1_-BSA is 74,355.301 Da, and the conjugation increases the protein molecular weight by 8144.33 Da (Figure 5a,c), thus the molecular coupling ratio of FB_1_ per BSA in conjugate is 11. The molecular weight of FB_1_-OVA is 48,009.212, and the incremental change is 3721.904 compared with OVA (Figure 5b,d), and the molecular coupling ratio of FB_1_ per OVA in conjugate is 5.

The method of MALDI-TOF-MS has been developed because the traditional approaches are often limited by sensitivity, selectivity or speed of method development [28]. The accuracy of this method is demonstrated by the coefficients for a TOF calibration function in a previous study [29]. Multiply-charged or cluster ions have different total kinetic energies when compared to their parent molecular ions.

To prepare an effective hapten-protein conjugate for the desired immune response, it is important to characterize the resulting hapten-protein conjugate to determine the hapten molecular coupling ratio on carrier protein (numbers of hapten molecules per carrier). The higher ratio of hapten usually increases the strength and specificity of the immune response. However, there is a risk that a high degree of substitution could adversely affect the activity and specificity of antibodies produced [30]. At present, no official method for the determination of hapten-protein is available. Generally, the evidence as to whether hapten has been successfully conjugated to the carrier protein is obtained after the putative conjugate is injected into laboratory animals and antibodies are detected, but this method is problematic, since it is time-consuming and wasteful [24]. Some methods have been tested in comparative studies [25,30], and UV is the technique commonly used for qualitation of the hapten-protein, including mycotoxins; however, this technique is not compatible in terms of the accuracy of FB_1_ due to the lack of ultraviolet absorption functional group. MALDI-TOF-MS allows the determination of FB_1_-protein with good accuracy and precision and is appropriate for determining the other hapten-protein [31].

## 3. Experimental Section

### 3.1. Chemicals and Regents

Fumonisin B_1_ (FB_1_, mw 721.8 Da, 98% purity), ovalbumin (OVA, mw 44,287 Da), glutaraldehyde solution 25% (W/V) were all purchased from Sigma-Aldrich (Shanghai, China). Bovine serum albumin (BSA, average mw 66 kDa) was obtained from Roche (Nanjing, China). Protein molecular weight standard (10–170 kDa) was obtained from Tianwei Biotechnology Co., Ltd. (Nanjing China). All chemicals, reagents, and solvents used in this study were of high purity analytical grade. Buffers were made in Milli-Q double distilled water.

### 3.2. Conjugation of Hapten with Protein

Two commercially available carrier proteins for modifying hapten, BSA and OVA, were tested for linking FB_1_ to produce the FB_1_-protein conjugates (FB_1_-OVA and FB_1_-BSA) in order to make its immunogenic. Due to its high efficiency, GA was used as cross-linker reagent for the reaction. The conjugation procedure was performed as previously described by F.Y. Yu, F.S. Chu [32], with minor modifications. In particular, the experiment was performed with a reagent molar ratio of 50:1 (FB_1_: protein). Protein solution (OVA or BSA) was prepared in phosphate-buffered saline (PBS, 0.01 M, pH 7.4) at concentration of 1 mg/mL. GA solution (25%, W/V) was diluted to 2% (W/V) with PBS. One milligram FB_1_ was place in a screw-cap amber vial and 1.8 mL of BSA solution, followed by the same volume of GA solution, were added to the vial. The vial was closed and mixed by magnetic agitation for 1 h at 4 °C. After the cap was removed, the powder of sodium borohydride (NaBH_4_) was added to the mixture (final concentration of 10 mg/mL), and the mixture was allowed to magnetic agitate for 1 h at 4 °C. The reaction solution was dialyzed for 72 h at 4 °C against ultra pure water, then, it was centrifuged for 15 min at 12,000 r/m to remove the precipitate. The supernatant was concentrated with ultrafiltration centrifugal tube (Millipore Amicon Ultra-4, Billerica, MA, USA) and stored at 4 °C. A portion of conjugation solution was placed in another 1.5-mL micro centrifuge tube and lyophilized with freeze-dryer (Labconco, Kansas, MO, USA).

### 3.3. Identification of FB_1_-Protein Conjugates by UV

The concentration of the new FB_1_-protein conjugate was determined by UV spectrophotometer (Tecan infinite M200, Seestrasse, Switzerland) at a wavelength of 280 nm. Moreover, full scan experiments (scan range 250 to 400 nm) were performed to analyze the changes of the absorption spectra and the characteristic absorbance contribution of the new FB_1_-protein conjugate. The samples were prepared as followed. One milligram of FB_1_ was dissolved in acetonitrile-water (50:50, v/v) (1.0 mg/mL) and the same concentrations of OVA and BSA were prepared. FB_1_-OVA and FB_1_-BSA were concentrated to the concentrations of 1.0 mg/mL, respectively. One hundred microliters of the reaction mixture were injected into the UV apparatus.

### 3.4. Identification of FB_1_-Protein Conjugates by FT-IR

In order to find the characteristic functional groups in FB_1_-protein conjugate, FT-IR full scan range from 4000 to 400 cm^−1^ were performed using an intelligent FT-IR spectrometer (Thermo NICOLET380, Waltham, MA, USA). The lyophilized powders were used in this test. One milligram of solid sample (FB_1_, OVA, BSA, FB_1_-OVA and FB_1_-BSA) was milled with potassium bromide (KBr) to form a very fine powder, respectively. This powder was then compressed into a thin pellet which could be analyzed. KBr was transparent in the IR. The mixture tablets were put into the intelligent FT-IR spectrometer. The changes of functional groups and structure before and after conjugation were analyzed.

### 3.5. Identification of FB_1_-Protein Conjugates by Gel Electrophoresis

The N-PAGE, N-AGE and SDS-PAGE of the conjugates and proteins were performed using Bio-Rad electrophoresis apparatus (Mini PROTEAN, Hercules, CA, USA) equipped with a Bio-Rid gel auto imaging system (Bio-Rid GelDocXR+, Hercules, CA, USA) and quantity one 1-D analysis software [33] to compare the difference of transport ratio.

The SDS-PAGE and N-PAGE procedure were performed as previously described by Sambrook [34]. In particular, 12% separated gel and 5% concentrated gel was used.

The modified procedure of N-AGE method was followed. Briefly, TAE buffer (40 mM tris-acetic acid, 2 mM EDTA) was employed for electrophoresis buffer and prepared at 4 °C. The loading buffer was prepared as followed, 20 mM EDTA, 0.04% (W/V) bromophenol blue (BPB) and 6.67% (W/V) sucrose. 1% agarose gel was 3–5 mm thick. Ten microliters of each sample (0.2 mg/mL), mixed with an equal volume of loading buffer, was applied to the gel and samples were separated at 240 V for 30 min in 4 °C. The gel was fixed with 20% trichloroacetic acid for 20 min, stained with Coomassie blue staining solution (0.1% W/V coomassie blue R-250, 25% V/V isopropanol, 10% V/V acetic acid) for 1 h, and then destained with destaining solution (10% V/V acetic acid, 5% V/V ethanol) with several changes until clear.

### 3.6. Identification of FB_1_-Protein Conjugates by MALDI-TOF-MS

MALDI-TOF-MS analysis was carried out using a MalDI-ToF/ToF/MS spectrometer (Bruker Ulfvaflex II, Bremen, Germany) to analyze the presence of molecular ions and the fragment ions for each conjugate. The molecular weight of each conjugate and the molecular coupling ratio of FB_1_ per protein were calculated, respectively. The lyophilized powders were used in this test. Gentisic acid (Sigma-Aldrich, Shanghai, China) was used as a matrix. The matrix solution was prepared as follows. Ten milligram of gentisic acid was dissolved in 1 mL of mixed solution of acetonitrile and 0.1% trifluoroacetic acid (TFA) (2:3, V/V). It is necessary to use a method of dropping to a sample plate that is suitable for different samples or matrices individually. The basic method procedure is described below. First, prepare an unused clean sample plate. Second, drop 0.5 μL of sample solution to the specified sample well using a pipette, *etc*. Take care not allow the pipette end to touch the plate. Third, drop a matrix solution onto 0.5 μL of sample solution before the crystallization of the sample (within approximately 10 s). Forth, air-dry. Caution: exchange the pipette tips every drop irrespective of matrices or sample solutions. The dropping order of a matrix and that of a sample may be reversed mutually.

## 4. Conclusions

In this comparing experiment of the six characterization method, MALDI-TOF-MS has been shown to be an efficient method due to its high accuracy and high performance in evaluating the cross-linking effect and calculate the molecular coupling ratio. The other methods, such as UV, FT-IR, MALDI-TOF-MS and gel electrophoresis are very useful in detecting for the cross-linking effect of hapten-protein conjugates intuitively. MALDI-TOF-MS is simple to perform and shows laboratory performances superior to other published methods for the determination of hapten-protein in terms of sensitivity, accuracy and precision.
